# Random Amplified Polymorphic DNA (RAPD) for differentiation between Thai and Myanmar strains of *Wuchereria bancrofti*

**DOI:** 10.1186/1475-2883-6-6

**Published:** 2007-07-30

**Authors:** Surang Nuchprayoon, Alisa Junpee, Yong Poovorawan

**Affiliations:** 1 Lymphatic Filariasis Research Unit, Department of Parasitology, Chulalongkorn Medical Research Center (Chula MRC), Chulalongkorn University, Bangkok 10330, Thailand; 2 Department of Pediatrics, Faculty of Medicine, Chulalongkorn University, Bangkok 10330, Thailand

## Abstract

**Background:**

Lymphatic filariasis (LF) is a mosquito-borne disease caused by mosquito-transmitted filarial nematodes, including *Wuchereria bancrofti *and *Brugia malayi*. The Lymphatic Filariasis Elimination Program in Thailand has reduced the prevalence of nocturnally subperiodic *W. bancrofti *(Thai strain), mainly transmitted by the *Ochlerotatus *(*Aedes*) *niveus *group in Thailand to 0.57/100,000 population. However, it is estimated that more than one million Myanmar migrants with high prevalence of bancroftian filariasis have settled in the large urban cities of Thailand. These infected migrants carry the nocturnally periodic *W. bancrofti *(Myanmar strain) which has *Culex quinquefasciatus *as the main mosquito vector. Although transmissions of the Myanmar strain of *W. bancrofti *by the Thai *Cx. quinquefasciatus *has never been reported, previous study showed that *Cx. quinquefasciatus *could nurture the Myanmar strain of *W. bancrofti *to the infective stage. Thus, the potential now exists for a re-emergence of bancroftian filariasis in Thailand. The present study was undertaken in an attempt to differentiate between the Thai and Myanmar strains of *W. bancrofti*.

**Methods:**

The microfilarial periodicity of Thai and the Myanmar strains of *W. bancrofti *were determined. Comparative morphology and morphometry of microfilariae and a study of random amplified polymorphic DNA (RAPD) was performed. The Nei's genetic distance was calculated, and a phylogenetic tree was constructed using the Unweighted Pair Group Method with Arithmetic mean (UPGMA).

**Results:**

The Thai strain of *W. bancrofti *was nocturnally subperiodic, and the Myanmar strain of *W. bancrofti *was nocturnally periodic. The body length, cephalic space length, and cephalic space width of the Thai strain of *W. bancrofti *were significantly larger than those of the Myanmar strain of *W. bancrofti *(p < 0.05). However, an overlapping mean of these parameters made it impractical for field application. RAPD-PCR profiles showed specific bands characteristic for the Myanmar strain of *W. bancrofti*. The phylogenetic tree indicated two genetically distinct clusters of the Thai and Myanmar strains of *W. bancrofti*.

**Discussion:**

This study was the first report on the genetic polymorphism of the Thai and Myanmar strains of *W. bancrofti*. Differentiation between the Thai and Myanmar strains of *W. bancrofti *could not rely on morphological criteria alone. However, RAPD profiles revealed a significant diversity between the two strains. The RAPD-PCR technique was suitable for differentiating Thai and Myanmar strains of *W. bancrofti*. The RAPD marker could be used for epidemiological assessment of the Myanmar strains of *W. bancrofti *in Thailand.

## Background

Lymphatic filariasis (LF), the second leading cause of long-term disability worldwide from lymphedema, elephantiasis, hydrocele and periodic fevers, is caused by mosquito-transmitted filarial parasites, *Wuchereria bancrofti *and *Brugia malayi *[1]. It is estimated that 1.1 billion people, 20% of the world's population, in more than 83 countries are at risk of acquiring the infection, while more than 120 million individuals have already been infected [2]. In 1993, the International Task Force for Disease Eradication (ITFDE) identified LF as one of six potentially eradicable infectious diseases. In 1997, the World Health Assembly passed a resolution to eliminate of LF as a public health problem by 2020 [3].

In Thailand, bancroftian filariasis is endemic on the Thai-Myanmar border (mainly Tak, Kanchanaburi, and Mae Hongson Provinces) [4-6]. The nocturnally subperiodic *W. bancrofti *(rural strain; Thai strain) found in infected Thai rural populations has the *Ochlerotatus *(*Aedes*) *niveus *group as the main mosquito vector. Recently, it has been reported that Myanmar migrant workers in Thailand carry *W. bancrofti *at a prevalence of 2–8% [4]. These infected Myanmar migrants carry the nocturnally periodic *W. bancrofti *(urban strain; Myanmar strain) which has *Culex quinquefasciatus *as the main mosquito vector [7]. *Cx. quinquefasciatus *readily breeds in urban areas of Thailand. The Thai strain of *Cx. quinquefasciatus *could nurture the Myanmar strain of *W. bancrofti *to the infective stage in a recent laboratory study [5]. This means that the urban Thai population is at risk of the infection. The high prevalence of *W. bancrofti *infection in Myanmar migrant workers has prompted concern that a re-emergence of bancroftian filariasis in Thailand is impending.

The Myanmar strain of *W. bancrofti *has been proven to be distinct from the Thai strain of *W. bancrofti *based on microfilarial periodicity, its dimensions and the number of nuclei between the cephalic space and nerve ring [8,9]. However, a morphological and morphometric study is time-consuming, laborious, and consequently not suitable for large-scale application. DNA polymorphism assay, based on random the amplified polymorphic DNA polymerase chain reaction (RAPD-PCR), has been proved useful for analyzing the inter- and intra-specific genetic variations and phylogenetic relationships. The RAPD technique is based on the amplification of a random DNA segment with a single primer of arbitrary nucleotide sequence and using the polymerase chain reaction [10,11]. This technique is very rapid, simple, and generates a reproducible fingerprint of the PCR products. In addition, it neither depends on previous knowledge or availability of the target DNA sequences nor requires DNA hybridization. The potential use of RAPD in taxonomy and population genetics has been widely documented [12-15], including with *W. bancrofti *populations in India [16,17]. However, no information is available concerning the genetic polymorphism between the Thai and Myanmar strains of *W. bancrofti*. We developed the RAPD-PCR technique to differentiate between the Thai and Myanmar strains of *W. bancrofti*.

## Methods

### Study population

**Figure 1:**
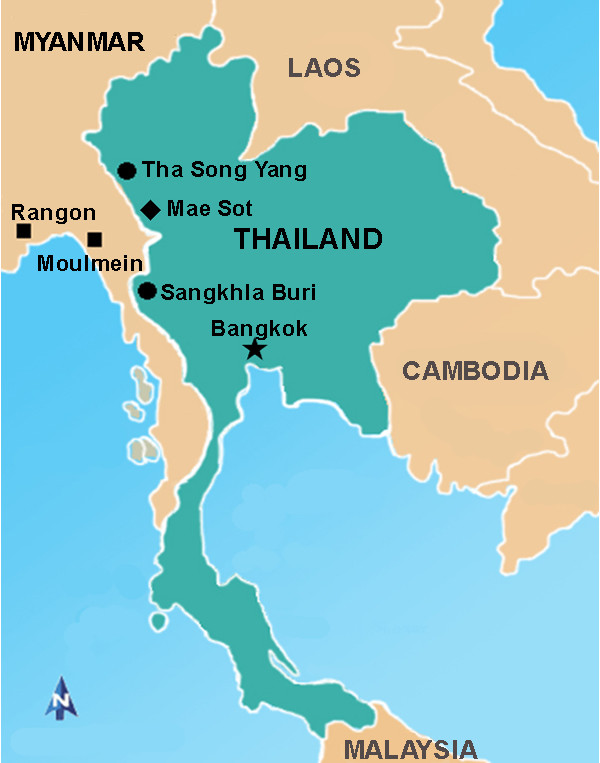
**Map of Thai-Myanmar border showing the study areas**. The Thai strain of *W. bancrofti *microfilariae are collected from patients living in Tha Song Yang District, Tak Province (solid circle); and Sankhla Buri District, Kanchanaburi Province (solid circle), Thailand. The Myanmar strain of *W. bancrofti *microfilariae are collected from Myanmar migrants living in Mae Sot District, Tak Province (solid diamond), Thailand. The Myanmar migrants were from Rangon and Moulmein (solid square), Myanmar.

The Thai strain of *W. bancrofti *microfilariae was collected from microfilaraemic Thai patients living in Tha Song Yang District, Tak Province, and Sankhla Buri District, Kanchanaburi Province, in the western region of Thailand. The Myanmar strain of *W. bancrofti *microfilariae were collected from microfilaraemic Myanmar migrants living in Mae Sot District, Tak Province, in the western region of Thailand. Among 6 Myanmar patients recruited for this study, 3 were from Rangon and 3 from Moulmein, Myanmar (Figure 1).

This study was reviewed and approved by the Ethics Committee of the Faculty of Medicine, Chulalongkorn University (Bangkok, Thailand). As almost none of the Myanmar workers spoke or read either Thai or English, verbal informed consent in the Myanmar language was obtained from each volunteer in the presence of two witnesses. We had a Thai-Myanmar interpreter for communicating with the Myanmar migrants. After the study was completed, all volunteers received a standard course of treatment with diethylcarbamacine.

### Microfilarial periodicity

The periodicity of each strain of *W. bancrofti *microfilariae was determined by counting the microfilariae from 20 μl finger-prick blood films, in triplicates, taken at 2-hour intervals over a period of 24 hours. We obtained microfilariae from 4 Thai and 4 Myanmar infected cases. Each blood film was stained with Giemsa (Merck, Darmstadt, Germany), as previously described [6,18,19]. The blood film was examined under a light microscope by two independent individuals. The average counts of microfilariae were plotted against time.

### Blood collection

Blood for morphological and morphometric studies and RAPD was obtained from each infected individual. Ten milliliters of venous blood were collected under sterile technique and universal precautions from Thai patients at 20.00 hours, and from Myanmar patients at 02.00 hours, as previously described [18,19].

### Morphological and morphometric study

The standard smear method [20] was used to prepare the slides. Twenty microliters of blood were smeared in a straight line on a clean slide, dried, dehemoglobinized, and stained with Giemsa. The stained microfilariae were assessed by counting the number of nuclei between cephalic space and nerve ring using a light microscope. The body length and width at various sites were measured using an ocular micrometer, the camera-lucida, and curve meter, determined by two independent individuals. Thirty microfilariae from each patient were studied. Differences in means were analyzed by standard *t*-test using SPSS software program.

### Microfilaria harvesting

One milliliter of blood was mixed with 9 ml of isotonic phosphate buffered saline (PBS [pH 7.4]; 137 mM NaCl, 2.7 mM KCl, 8 mM Na2HPO4, and 1.5 mM KH2PO4), filtered through a 5.0 μM Millipore membrane filter (Millipore, Billerica, MA), followed by adding approximately 20 ml of PBS, and air drying [20]. After the filters were transferred to the plate containing PBS, microfilariae were harvested using a needle under a stereo microscope, and suspended in 10 μl of PBS.

### DNA extraction

DNA extraction was performed using FTA paper (Whatman Bioscience, Cambridge, UK) as previously described [21]. Briefly, the microfilariae were washed twice with PBS, and then subjected to 3 freeze-thaw cycles, and blotted onto the FTA paper. After air drying, the FTA paper was washed twice with 200 μl of FTA purification buffer (Life Technologies, Gaithersburg, MD) for 15 min, washed twice with 200 μl of TE^-1 ^buffer (10 mM Tris-HCl [pH 8.0], 0.1 mM EDTA [pH 8.0]) for 5 min, and then dried on a heating block at 56°C for 10 min. The dried FTA paper was used as the DNA template in RAPD-PCR amplification.

### RAPD-PCR parameters

Six oligonucleotides from the ready-to-go RAPD analysis kit (Amersham Biosciences Ltd., Piscataway, NJ) were used for amplification of random DNA markers to reveal the genetic diversity among *W. bancrofti *populations. RAPD-PCR was performed with ready-to-go RAPD analysis Beads as described by the manufacturer (Amersham Biosciences). Approximately 10 ng of genomic DNA from microfilriae was used. The RAPD reaction was performed in a DNA thermal cycler (GeneAmp PCR System 2400, Perkin-Elmer, Norwalk, CT), for 1 cycle at 96°C for 4 min, followed by 40 cycles of 94°C for 1 min, 40°C for 1 min and 72°C for 2 min, respectively. The final amplification cycle included 7 min extension at 72°C. Amplified products were analyzed by 2% agarose gel (USB, Cleveland, OH) electrophoresis, stained with ethidium bromide, and visualized under ultraviolet light. The size of each band was determined by Quantity One^® ^1-D Analysis Software (Bio-Rad, Hercules, CA). RAPD analysis of human DNA (control), using the same primer and protocol was also conducted to rule out the possibility of contamination of human DNA in the samples. The amplification of all the DNA samples was repeated three times in order to see the variability, if any, in the amplification patterns. Similar patterns were obtained from all experiments.

### Phylogenetic analysis of the RAPD profiles

RAPD profiles were used to measure genetic similarity among *W. bancrofti *populations. The presence or absence of bands was coded in binary (0, 1) form in a data matrix. Parasites from one individual patient were treated as a genetic population. Relationships among the individual genetic populations of *W. bancrofti *were determined by a distance matrix method. The approach involved calculation of the Nei (1973) index of genetic similarity [22]. Distance values were subjected to phylogenetic analyses using the Unweighted Pair Group Method with Arithmetic mean (UPGMA) as implemented in the UPGMA program of the PAUP software package, version 4.0b10 [23].

## Results

### Microfilarial periodicity

**Figure 2:**
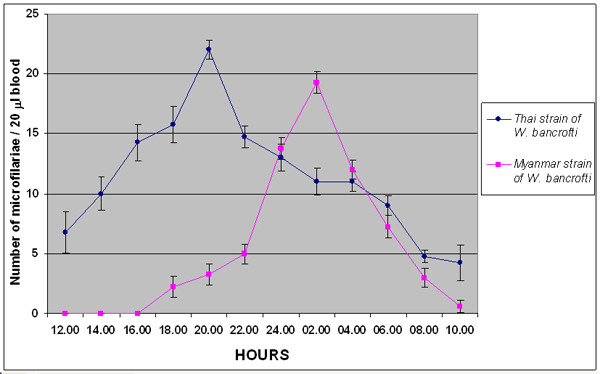
**Microfilarial periodicity of the Thai and Myanmar strains of *Wuchereria bancrofti***. The periodicity profile of nocturnally subperiodic *W. bancrofti *(Thai strain) in four Thai patients, and nocturnally periodic *W. bancrofti *(Myanmar strain) in four Myanmar patients based on the average counts of microfilariae in triplicated of 20 μl blood samples. Values shown are means ± SD.

The periodicity profiles of the Thai and Myanmar strains of *W. bancrofti *were determined (Figure 2). The microfilariae were present in peripheral blood of 4 Thai patients at all times during a 24-hour period but with higher numbers during the nighttime than during the daytime. This indicated the nocturnal supperiodicity character of Thai strain of *W. bancrofti*. The peak count of the Thai strain of *W. bancrofti *microfilariae in peripheral blood was at 20.00 hours.

The microfilariae were present in peripheral blood of 4 Myanmar patients during 18.00 hours and 10.00 hours, and absent during 12.00 hours and 16.00 hours, indicating the nocturnal periodicity character of the Myanmar strains of *W. bancrofti*. The peak count of the Myanmar strain of *W. bancrofti *microfilariae in peripheral blood was at 02.00 hours.

### Microfilarial morphology and morphometry

Microfilariae of both the Thai and Myanmar strains of *W. bancrofti *were sheathed, lying in graceful coils without secondary kinking. Somatic nuclei were discrete, overlapping where crowded but with distinct borders, and countable. The nuclear column stopped before the tip of the tail.

Morphometric measurements and counts of nuclei between the cephalic space and nerve ring of the two strains of *W. bancrofti *microfilariae were demonstrated (Table 1). Some measurement parameters of the Thai strain of *W. bancrofti *(body length, 299.20 ± 11.87 μm; cephalic space length, 5.47 ± 0.68 μm; and cephalic space width, 5.91 ± 0.53 μm) were significantly (p < 0.05) larger than those of the Myanmar strain of *W. bancrofti *(the body length, 286.92 ± 9.42 μm; cephalic space length, 5.20 ± 0.71 μm; and cephalic space width, 5.62 ± 0.57 μm). However, other parameters of the Thai strain of *W. bancrofti*, including head to nerve ring length, 56.81 ± 5.33 μm; Innenkorper length, 42.62 ± 5.79 μm; body width at nerve ring, 6.32 ± 0.52 μm; and number of nuclei between cephalic space and nerve ring, 89.52 ± 6.37, were not statistically different from those of the Myanmar strain of *W. bancrofti *(head to nerve ring length, 56.24 ± 3.26 μm; Innenkorper length, 42.67 ± 5.09 μm; body width at nerve ring, 6.30 ± 0.69 μm; and number of nuclei between cephalic space and nerve ring, 88.51 ± 8.29).

**Table 1:** Morphometric measurements and nuclei counts between the cephalic space and nerve ring of the Thai and Myanmar strains of *Wuchereria bancrofti *microfilariae

	*Wuchereria bancrofti *strains**		
		
Parameters*	**Thai (nocturnally subperiodic)**	**Myanmar (nocturnally periodic)**	***t*, (*p*)**
**Measurements (μm)**			
Body length	299.20 ± 11.87 (270–324)	286.92 ± 9.42 (255–310)	8.879, (0.000***)
Cephalic space length	5.47 ± 0.68 (4–7)	5.20 ± 0.71 (4–7)	-2.945, (0.004***)
Head to nerve ring length	56.81 ± 5.33 (34–72)	56.24 ± 3.26 (48–67)	0.994, (0.321)
Innenkorper length	42.62 ± 5.79 (32–64)	42.67 ± 5.09 (32–60)	-0.710, (0.943)
Cephalic space width	5.91 ± 0.53 (4–8)	5.62 ± 0.57 (4–7)	-4.097, (0.000***)
Body width at nerve ring	6.32 ± 0.52 (6–8)	6.30 ± 0.69 (5–9)	0.211, (0.833)
**Count**			
Number of nuclei between cephalic space and nerve ring	89.52 ± 6.37 (72–103)	88.51 ± 8.29 (66–101)	-1.100, (2.72)

* Measurements and count: mean ± SD. Range in parentheses.

** Thirty samples from each patient, total 120 samples per strain.

*** Statistically significant difference at *p *< 0.05

### RAPD analysis

**Figure 3:**
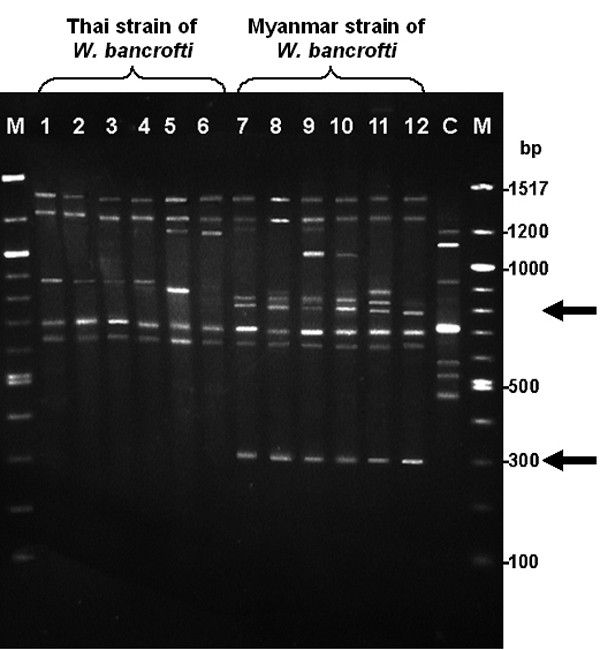
**Random Amplified Polymorphic DNA (RAPD) profile of Thai and Myanmar strains of *Wuchereria bancrofti***. The RAPD profile of the Thai and Myanmar strains of *W. bancrofti *using Primer 1 (5'-dGGTGCGGGAA-3'): Lane M: 100 bp DNA marker; Lane 1–6: the Thai strain of *W. bancrofti*; Lane 7–12: the Myanmar strain of *W. bancrofti*; Lane C: Negative Control (uninfected human blood sample). The 300 bp and 795 bp fragments (arrows) are specific to the Myanmar strain of *W. bancrofti*. The 645, 705, 1290, and 1400 bp fragments were common in both the Thai and Myanmar strains of *W. bancrofti*.

Out of the six oligonucleotides assayed, only the RAPD Analysis Primer 1 (5'-dGGTGCGGGAA-3') revealed unambiguous genetic polymorphisms between the Thai and Myanmar strains of *W. bancrofti*, and was retained for the analysis in this study. The RAPD profiles of *W. bancrofti *from 12 genetic populations (6 Thai patients and 6 Myanmar patients) were analyzed (Figure 3). The analysis of RAPD profiles indicated the existence of genetic variability between the Thai and Myanmar strains of *W. bancrofti*. The number of DNA fragments amplified in the RAPD-PCR from each sample ranged from 5–9. The fragment sizes of different bands recorded for all the samples ranged from 300–1400 bp. The 300 bp and 795 bp bands were specific to the Myanmar strain of *W. bancrofti *because the Thai strain did not show these bands. Both strains shared the 645, 705, 1290, and 1400 bp bands. Amplification of human DNA (non-infected with *W. bancrofti*) with the same primer, under similar conditions, yielded 7 fragments ranging from 470 to 1200 bp, but none of these fragments was identical to the fragments amplified in the *W. bancrofti *DNA samples (Figure 3). This observation shows that there was no human DNA contamination in the parasite DNA samples used for this study.

### Phylogenetic analysis

**Figure 4:**
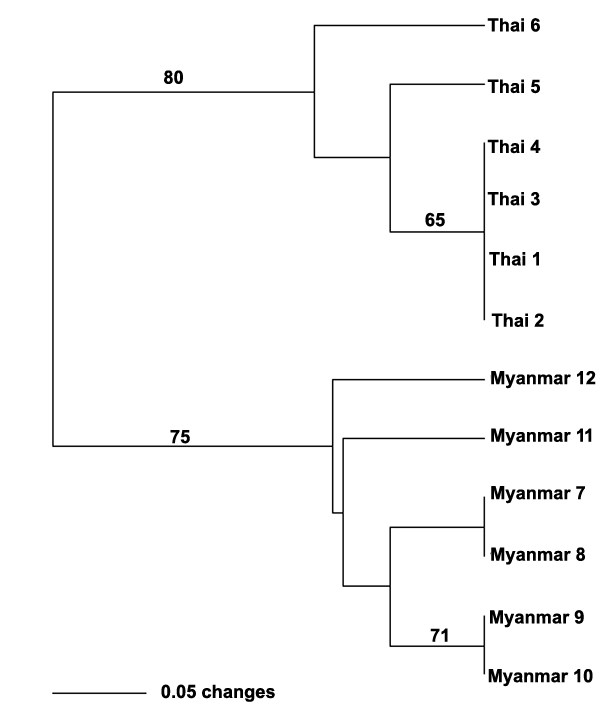
**Phylogenetic tree using UPGMA of individual population of *Wuchereria bancrofti***. The phylogenetic tree representing the relationships between the Thai and Myanmar strains of *W. bancrofti *constructed by PAUP software. Bootstrap values are shown above the branches where greater than 50% obtained after 1000 replicates. Thai 1-Thai 6: genetic populations of the Thai strain of *W. bancrofti*; Myanmar 7-Myanmar12: genetic populations of the Myanmar strain of *W. bancrofti*.

A phylogenetic tree was constructed using PAUP software to characterize *W. bancrofti *populations through phylogenetic analysis. Twelve genetic populations fell into two major clusters (Figure 4). The upper half of tree consisted of 6 genetic populations from the Thai patients. The lower branch consisted of all 6 genetic populations from the Myanmar patients. This phylogenetic tree demonstrated clearly that a genetic diversity existed between the Thai and Myanmar strains of *W. bancrofti*.

## Discussion

The microfilarial periodicity of the Thai strain of *W. bancrofti *was nocturnally subperiodic, with a peak microfilaremia at 20.00 hours (Figure 2). The microfilarial periodicity of the Myanmar strain of *W. bancrofti *was nocturnally periodic, with a peak microfilaremia at 02.00 hours. The microfilarial periodicity found in our study corresponded to previous reports [8,9].

We found that only 3 morphometric measurement parameters, body length, cephalic space length, and cephalic space width, of the Thai strain were significantly larger than those of the Myanmar strain of *W. bancrofti *(Table 1), while previous studies [8,9] showed that all parameters measured in the Thai strain of *W. bancrofti *were larger than in the Myanmar strain of *W. bancrofti*. This might be the result of a difference of geographic locations of the study populations. The Thai strain of *W. bancrofti *in our study was obtained from Tha Song Yang District, Tak Province, and Sankhla Buri District, Kanchanaburi Province, while the Thai strain of *W. bancrofti *in previous studies was obtained from Mae-Ramat District, Tak province. However, the previous studies do not provide the information where the Myanmar strain of *W. bancrofti *was obtained. Further studies would clarify this issue. Although the body length, cephalic space length, and cephalic space width were significantly different between the Thai and Myanmar strains of *W. bancrofti*, the ranges of these parameters were overlapping (body length, 270–324 μm *versus *255–310 μm; cephalic space length, 4.00–7.00 μm *versus *4.00–7.00 μm; and cephalic space width, 4.00–8.00 μm *versus *4.00–7.00 μm, from the Thai strain and the Myanmar strains of *W. bancrofti*, respectively). The overlapping range of values made it impractical to differentiate between the Thai and Myanmar strains of *W. bancrofti*. In addition, the morphological and morphometric studies were time-consuming, laborious, and consequently not suitable for large-scale application [8,9]. This difference can only be distinguished by highly experienced personnel [8,9].

Differentiation of filarial nematodes have been reported, using DNA hybridization assays [24-27], PCR [18,28-30], PCR-RFLP [6,31-34], and RAPD [12-17]. However, there is no report of any molecular technique that could differentiate between the Thai and Myanmar strains of *W. bancrofti*. Our RAPD profiles of the Thai and Myanmar strains of *W. bancrofti *revealed a significant diversity (Figure 3), with 300 bp and 795 bp bands specific for the Myanmar strain of *W. bancrofti*. The phylogenetic analysis exhibited two distinct clusters of the Thai and Myanmar strains of *W. bancrofti *(Figure 4). Therefore RAPD-PCR was suitable to differentiate between the Thai and Myanmar strains of *W. bancrofti*. The RAPD-PCR has been proven to be an easy, reproducible and rapid technique that could be used as a diagnostic tool to assess the real burden of Thai and Myanmar strains of *W. bancrofti *in Thailand. Further study of the Myanmar strain of *W. bancrofti*-specific bands by Sequence Characterized Amplified Region (SCAR) [35,36] should be performed to develop strain-specific PCR primers/probes, to identify the Myanmar strain of *W. bancrofti*.

## Conclusion

Although the existence of different strains of *W. bancrofti *(based on microfilarial periodicity) was documented in the past [20], differentiation techniques using laboratory methods (eg. morphological and morphometric study) have been a problem. We could differentiate between the Thai and Myanmar strains of *W. bancrofti *by comparing morphology and morphometry using microscopy, biological behavior by microfilarial periodicity, and genetics by RAPD technique. However, the PCR-based technique for RAPD could clearly characterize genetic differences between the Thai and Myanmar strains of *W. bancrofti*. The RAPD-PCR technique was indeed useful for the differentiation of strains of *W. bancrofti *because of its relative ease, simplicity and reproducibility.

## Competing interests

The author(s) declare that they have no competing interests.

## Authors' contributions

SN had primary responsibility for all studies (planning, execution of the experiments, data analysis, and writing); AJ collected samples, conducted RAPD-PCR assays, data entry and data analysis, and YP assisted with fieldwork design. All authors have read and approved the final manuscript.
